# A pilot-wellness-informed ecological framework for human-autonomy teaming in future fighter aviation

**DOI:** 10.3389/fphys.2026.1913172

**Published:** 2026-09-30

**Authors:** Caelan Midwood

**Affiliations:** Defence Research and Development Canada, Suffield Research Centre, Medicine Hat, AB, Canada

**Keywords:** adaptive automation, digital twin, future fighter, human-autonomy teaming, pilot state monitoring, situation awareness, systems science

## Abstract

Future fighter operations are expected to combine increasingly capable autonomy, collaborative aircraft, dense sensor information, and sustained physiological and cognitive demands. This Perspective proposes a pilot-wellness-informed ecological framework in which the pilot, a pilot-specific digital twin, an intelligent flight-management system, the aircraft, and external agents are treated as interdependent elements within a systems-of-systems framework. The framework does not replace situation awareness, distributed cognition, joint cognitive systems, adaptive automation, or human-autonomy teaming. Instead, it extends their unit of analysis by making the pilot’s dynamic physiological and neurocognitive state, longitudinal variability, and health-related constraints explicit system variables and design objectives. The pilot digital twin is defined narrowly as an individualized state-estimation and forecasting capability, whereas the intelligent flight-management system integrates pilot, platform, mission, and environmental information to provide explainable recommendations and, where authorized, bounded adaptive action. We identify testable propositions concerning individualized prediction, adaptive support, shared situation awareness, trust calibration, and governance. The framework is prospective and requires validation in simulation, training, and operationally representative environments. Its purpose is to organize research toward systems that protect pilot agency, sustain performance, and preserve long-term health while exploiting increasingly autonomous teammates.

## 1 Introduction

Fighter generations summarize broad changes in survivability, sensing, connectivity, propulsion, and weapons integration, but they are not standardized scientific classifications and future configurations remain uncertain (Hallion, 1990; Shklarsky and Shamir, 2023). Future operations are nevertheless likely to combine a crewed aircraft, advanced decision support, collaborative combat aircraft, distributed command-and-control, and persistent data exchange. The pilot may consequently shift from direct vehicle control toward mission command, supervision of autonomous assets, interpretation of fused information, and negotiation of authority across a human-machine team.

These changes may reduce manual demands while redistributing cognitive and physiological workload. Automation can create mode confusion and leave operators responsible for rare, time-critical interventions (Bainbridge, 1983; Sarter and Woods, 1995). Fighter pilots also operate under acceleration, hypoxia risk, thermal strain, fatigue, vibration, sensory loading, and time pressure. Physiological and behavioral measures can provide complementary information about workload, engagement, fatigue, and situation awareness, but findings show marked inter-individual variability and limited transfer from controlled to operational environments (Borghini et al., 2014; Singh et al., 2021; van Weelden et al., 2022; Shaw and Harrell, 2023; Richardson et al., 2025; Hamann et al., 2026; Sekkati et al., 2026).

Mature human-factors frameworks already address much of this problem. Situation-awareness theory describes perception, comprehension, and projection in dynamic environments (Endsley, 1995). Distributed cognition and joint cognitive systems locate cognition across people, artifacts, procedures, and environments (Hutchins, 1995; Hollnagel and Woods, 2005). Models of interaction with automation distinguish information acquisition, analysis, decision selection, and action implementation (Parasuraman et al., 2000); adaptive-automation frameworks specify changes in task allocation or interface behavior (Feigh et al., 2012; Bernabei and Costantino, 2024); and human-autonomy teaming emphasizes roles, coordination, transparency, shared mental models, and mutual predictability (Klein et al., 2004; Cooke et al., 2013; Lyons et al., 2021; Tokadli and Dorneich, 2023; Stensrud et al., 2024).

Hence, the need is to integrate system boundaries that treat pilot wellness as a persistent, individualized, and safety-relevant entity. Operator-state monitoring is often framed as a sensor or interface problem, teaming at the level of task performance, and digital twins around equipment or maintenance. Future fighters require these perspectives to be connected across control actions, minute-to-minute workload, training adaptation, recovery, and cumulative health. Without an explicit relational vocabulary, physiological estimates can be discussed without specifying who receives them, how uncertainty is represented, or what authority they can trigger.

This Perspective proposes a pilot-wellness-informed ecological framework for that integration. Ecology is used as an ontology and systems-analysis heuristic for entities, relationships, feedback, constraints, adaptation, and emergent behavior; it is neither a biological classification of AI nor a replacement for established theory. The framework contributes an expanded boundary containing the pilot’s biological state, an individualized pilot digital twin (PDT), an intelligent flight-management system (IFMS), the aircraft, and external agents; a functional separation of estimation, prediction, recommendation, authority, and execution; and testable propositions addressing performance, safety, trust, privacy, certification, and long-term health.

## 2 Theoretical foundations and the added value of an ecological perspective

### 2.1 Established human-factors foundations

Situation awareness remains central because pilots must perceive relevant cues, understand their meaning, and project future states (Endsley, 1995). In a human-autonomy team, the pilot must also understand system capabilities, limitations, mode, intended actions, and confidence, while autonomy requires usable models of pilot intent, attention, workload, and capacity. This calls for coordinated rather than disconnected situation awareness. Transparency is useful when it conveys what the system knows, why it recommends an action, what it will do next, and how uncertain it is; indiscriminate disclosure can add workload (Endsley, 2023).

Distributed-cognition and joint-cognitive-system perspectives describe operational cognition as an achievement of humans, technologies, representations, procedures, and the work environment (Hutchins, 1995; Hollnagel and Woods, 2005). The relevant unit is the coupled system that maintains control. Interactive team cognition likewise treats cognition as communication and coordination activity, not only overlapping internal knowledge (Cooke et al., 2013).

Automation may execute predetermined functions with varying human involvement, whereas autonomy selects among courses of action under uncertainty with some goal-directed independence (Parasuraman et al., 2000; Lyons et al., 2021). Capability alone does not make a teammate: team participation requires interpredictability, common ground, directability, coordinated adaptation, explicit roles, delegation mechanisms, interaction paradigms, and training (Klein et al., 2004; Tokadli and Dorneich, 2023).

Adaptive automation changes task allocation, information presentation, modality, or authority in response to operator, system, environmental, mission, or temporal conditions (Feigh et al., 2012; Hussein et al., 2022). It may manage workload but can also degrade situation awareness or create unexpected transitions. The objective is a predictable, reversible distribution of work, not maximum autonomy.

### 2.2 Ecology as ontology and research heuristic

In this study, an operational ecology is defined as a bounded network of biological, technical, computational, and organizational elements whose states and relationships influence the capacity of the whole system to pursue shared goals. This use of ecology has four implications.

First, relationships are primary analytical objects: a physiological measurement has no operational meaning without sensing, a state model, uncertainty, an authorized recipient, and defined responses. Second, effects are reciprocal; autonomy may reduce workload but also engagement, and an attention alert may increase stress. Third, relevant time scales range from flight-control events to mission task allocation, readiness, training, and cumulative health. Fourth, outcomes such as calibrated trust, shared situation awareness, resilient coordination, and preserved health emerge from interactions and cannot be assigned to one component.

The ecological perspective links rather than replaces established frameworks. Situation-awareness theory defines relevant pilot and team states; joint cognitive systems define the unit of analysis; adaptive automation specifies changes in function allocation; and human-autonomy teaming supplies principles for roles, communication, trust, and coordination. The added contribution is their explicit integration with individualized physiology and longitudinal health context.

No new taxonomic category is required for AI. Components can instead be described by observable attributes: persistence, information access, state estimation, learning or adaptation, goal-directed decision-making, authority, and physical effect. This avoids implying that an AI component is biologically alive or equivalent to a human teammate.

## 3 Entities, functions, and boundaries

The proposed system contains five principal entity classes: the pilot, PDT, IFMS, aircraft, and external agents. Their functional boundaries are summarized in **Table 1** and their relationships in **Figure 1**.

**Table 1:** Functional allocation within the proposed pilot-wellness-informed system.

Entity	State represented	Functional allocation and authority
Pilot	Intent, goals, subjective experience, professional judgment, and self-state	Interprets the situation and self-state; anticipates consequences; accepts, rejects, or modifies options; retains mission command, authorization, override, and direct-control authority.
Pilot digital twin (PDT)	Individual physiological and neurocognitive state	Estimates and forecasts pilot-state trajectories with uncertainty; supplies confidence-weighted constraints and alerts; has no direct command or execution authority by default.
Intelligent flight-management system (IFMS)	Mission, team, task, and platform state	Fuses joint-system state; predicts mission and team consequences; generates explainable options and adaptive-support proposals; commands only within a bounded, pre-authorized authority envelope.
Aircraft	Vehicle, sensor, control, cockpit, and life-support state	Measures platform and cockpit variables; provides alerts and envelope information; executes valid pilot or IFMS commands and certified safety protections through controls and effectors.
External agents	Shared mission, environmental, and agent state	Estimate and predict assigned-domain outcomes; provide task or coordination proposals; execute assigned actions under authority defined by mission rules and rules of engagement.

**Figure 1:**
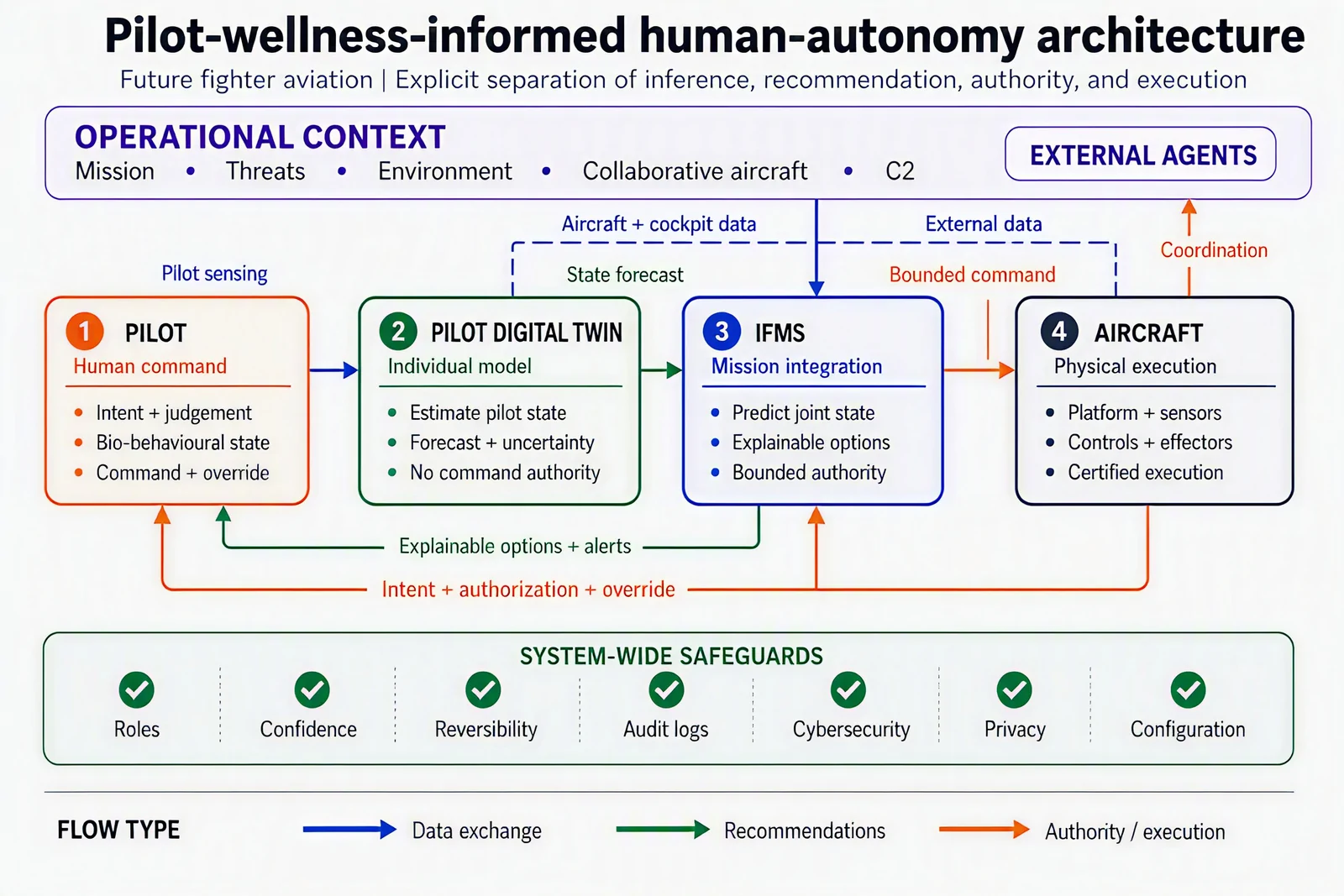
Pilot-wellness-informed architecture for future fighter human–autonomy teaming. The framework separates pilot-state inference, mission-level integration and recommendation, decision authority, and physical execution across four interacting components (1): the pilot, who retains mission intent, professional judgment, authorization, and override authority, except for explicitly pre-authorized safety functions (2); the Pilot Digital Twin (PDT), which integrates physiological, behavioral, and performance data to estimate and forecast pilot state and associated uncertainty, providing confidence-weighted forecasts, constraints, and alerts without direct command authority (3); the Intelligent Flight-Management System (IFMS), which integrates PDT outputs with aircraft, cockpit, mission, environmental, and external-agent information to predict joint-system consequences, generate explainable options, and initiate bounded adaptive actions within a certified authority envelope; and (4) the aircraft, which provides platform and sensor-state information and executes authorized commands through its controls, effectors, and certified safety protections. Blue dashed pathways represent sensing and data exchange, green pathways represent recommendations, forecasts, alerts, and feedback, and orange pathways represent authorization, command, coordination, and execution. System-wide safeguards—including role clarity, confidence thresholds, reversibility, event logging, cybersecurity, privacy, and configuration control—govern interactions throughout the architecture.

### 3.1 The pilot

The pilot is the biological operator, mission commander, and bearer of ethical and professional accountability. Relevant state is multidimensional and time-varying, including workload, attention, fatigue, stress, orientation, sensory function, cardiorespiratory status, acceleration tolerance, and thermal strain. These dimensions cannot be collapsed into a universal readiness score; they require context-specific constructs, validated measures, individual baselines, and uncertainty estimates.

Pilot authority should remain explicit. The pilot supplies mission intent, authorizes or rejects recommendations, directs autonomous teammates, and can override behavior within aircraft-safety limits. Pre-authorized emergency exceptions may be justified when response time is insufficient, but they require certification, transparent annunciation, bounded action, and auditability.

### 3.2 The pilot digital twin

A digital twin is a computational representation of a physical referent, updated through observations and used for state estimation, simulation, or prediction (Barricelli et al., 2019; Rasheed et al., 2020). The PDT is defined narrowly as an individualized, dynamically updated model of pilot state and response. It can combine baseline information, in-flight physiology and behavior, task context, environmental exposure, prior responses, and model confidence to estimate current state and forecast near-term change.

The PDT is not an autonomous pilot, flight controller, clinical diagnostic system, or bearer of command authority. It is an inference and forecasting service whose outputs specify the construct, time horizon, confidence, salient contributing factors where interpretable, and invalidating conditions. It might forecast overload during a task sequence or report inadequate sensor quality; it should not convert an uncertain pattern into an unqualified declaration of pilot fitness.

Individualization is scientifically demanding because physiological associations vary across people, tasks, environments, and time. Models using random cross-validation may fail prospectively or under distribution shift (Singh et al., 2021). A PDT therefore requires longitudinal calibration, external validation, drift monitoring, conservative thresholds, and graceful degradation when data are missing or unreliable.

### 3.3 The intelligent flight-management system

The IFMS integrates aircraft state, mission goals, environmental and external-agent information, pilot inputs, and confidence-weighted PDT outputs. It may detect conflicts, generate courses of action, predict mission consequences, prioritize information, recommend task reallocations, and coordinate collaborative aircraft.

The PDT predicts aspects of the pilot; the IFMS predicts and manages aspects of the mission and joint system. The PDT does not command the aircraft. The IFMS may command only within a certified authority envelope and should preserve a traceable chain from input to recommendation, authorization, execution, and outcome. Adaptive functions should be graded and reversible where conditions permit.

### 3.4 Aircraft and external agents

The aircraft comprises the platform, sensors, effectors, life support, displays, controls, and embedded computing. It provides platform state, cockpit exposures, control status, and sensor-quality information and executes pilot commands and authorized IFMS actions. It is not the source of pilot physiology: cockpit sensors acquire pilot signals and route them to estimation functions.

External agents include collaborative combat aircraft, command-and-control, other crewed platforms, intelligence, weather, maintenance, and support systems. Their data can change workload, threat assessment, task allocation, and authority; latency, link degradation, cyber compromise, or divergent objectives can alter the behavior of the whole team.

## 4 The pilot-wellness-informed adaptive loop

The framework forms a closed but governable loop. Time-synchronized physiological, behavioral, performance, aircraft, and environmental signals are acquired with quality indicators. The PDT then estimates defined pilot states and forecasts them with uncertainty. The IFMS combines those estimates with task demands, platform status, mission priorities, and external information, then selects a response within its authority. Responses can range from monitoring, information reprioritization, or interface simplification to task reallocation, autonomous support, or a pre-authorized safety action. Observed consequences are used to evaluate effects on pilot state, coordination, safety, and mission performance.

Not every detected change warrants intervention. Physiological signals are indirect and nonspecific: heart rate, electroencephalography, and ocular behavior can reflect several processes. Multimodal sensing does not remove the need for construct validity, temporal alignment, individual calibration, and context (van Weelden et al., 2022; Hamann et al., 2026; Sekkati et al., 2026). Confidence-weighted estimates and conservative thresholds are preferable to categorical labels.

Adaptation should follow a least-intrusive-effective-response principle. Weak evidence may justify monitoring or confirmation; moderate confidence, noncritical information simplification or task deferral; and strong evidence with severe consequences, a recommended task transfer or a previously authorized safety mode. Transitions should be predictable, visible, reversible when feasible, and recorded.

The optimization target is not minimum workload. Too little workload can reduce engagement, whereas intrusive assistance can increase demand or erode trust. The target is a dynamic region in which the pilot retains capacity, situation awareness, and authority while the team meets mission objectives without avoidable acute or cumulative physiological strain.

## 5 Human–AI coordination, cognition, and trust

Trust is necessary but insufficient for effective human-autonomy teaming. The desired state is calibrated trust: reliance that is appropriately matched to system capability, uncertainty, and context (Lee and See, 2004; Hoff and Bashir, 2015). Under-trust can lead to disuse and unnecessary workload; over-trust can produce automation bias, reduced monitoring, and delayed intervention. Trust will be shaped by reliability, predictability, transparency, prior experience, training, and the perceived legitimacy of the system’s access to personal physiological data.

Cockpit systems must support shared mental models, directability, mutual predictability, and common ground. The pilot should understand the IFMS goal, evidence, expectation, and intended action; the IFMS should receive mission intent, respect authority boundaries, identify ambiguity, and request clarification. Under combat time constraints, recommendation, rationale, confidence, and next action may be more useful than exhaustive explanation.

Authority negotiation is especially important when pilot-state estimates influence automation. A system that abruptly assumes control because it infers overload may create surprise, threaten professional identity, and undermine subsequent reliance. Conversely, a system that never acts without a lengthy confirmation may provide little value during a rapid incapacitation event. Authority should therefore be established through scenario-specific rules, training, and certification. Advisory, consent-based adaptive, and pre-authorized emergency modes should be visibly distinct.

Training should develop team skills, including experience with uncertainty, false alarms, missed detections, sensor or communication loss, conflicting recommendations, and automation recovery. The PDT and IFMS require evaluation across representative pilots and contexts; personalization cannot justify unvalidated online learning. Simulation can calibrate models, develop shared expectations, and exercise rare failures before operational use.

## 6 Testable propositions and research agenda

The ecological framework is useful only if it generates discriminable predictions and design requirements. Five propositions are offered for empirical evaluation.

### 6.1 Proposition 1: individualized state models will outperform population-only thresholds

For defined outcomes and time horizons, models incorporating within-pilot baselines and longitudinal responses should predict pilot-state transitions more accurately and with better calibration than models using population thresholds alone. This proposition should be tested prospectively, with temporal and participant-level separation between training and validation data. Outcomes should include discrimination, calibration, false-alert burden, time-to-detection, and robustness under sensor loss and distribution shift.

### 6.2 Proposition 2: pilot-state-informed adaptation will outperform mission-state-only adaptation under selected conditions

In scenarios where task demand interacts with fatigue, workload, hypoxia, acceleration, thermal strain, or other validated states, combining pilot-state estimates with mission and platform information should improve joint-system performance compared with adaptation based only on mission state. Relevant outcomes include errors, response time, mission effectiveness, workload, situation awareness, physiological recovery, and unnecessary intervention. The proposition does not predict universal benefit; it predicts benefit only when the inferred state is valid, actionable, and connected to an effective response.

### 6.3 Proposition 3: explicit roles and confidence-aware explanations will improve calibrated reliance and shared situation awareness

Interfaces that distinguish PDT inference, IFMS recommendation, pilot authorization, and system execution, and that communicate uncertainty at the appropriate level of detail, should reduce mode confusion and improve prediction of system behavior. Experimental comparisons should measure trust calibration rather than trust alone, along with situation awareness, intervention timing, communication efficiency, and recovery after system error.

### 6.4 Proposition 4: reversible, graded adaptation will produce greater resilience than binary transfer of control

Adaptive support that escalates through predefined and reversible levels should preserve engagement and facilitate recovery better than abrupt all-or-none transitions, except where the time-to-harm requires immediate action. Studies should manipulate urgency, automation reliability, and locus of authority while evaluating workload, startle, takeover performance, and post-event trust.

### 6.5 Proposition 5: governance will determine acceptance and operational utility

Pilots should be more willing to use physiological monitoring when its purpose, access rules, retention, uncertainty, and effects on flight status are explicit and independently governed. This proposition is particularly relevant because military pilots may avoid health care when they fear loss of flying status (Hoffman et al., 2023). Research should evaluate consent and acceptance under alternative governance models, including strict separation between real-time mission support and aeromedical or administrative use.

A staged research program should begin with construct definition and sensor validation, progress through retrospective and prospective model development, and then evaluate closed-loop adaptation in simulation. High-fidelity and operationally representative studies should follow only after performance, calibration, human-factors, cyber, and safety criteria are met. Test programs should include normal operations, edge cases, adversarial or corrupted inputs, communication degradation, sensor failure, and model drift. Both individual and team-level outcomes are required because an intervention that improves a single pilot-state metric may still degrade shared situation awareness or mission performance.

## 7 Safety, certification, ethics, and governance

Highly adaptive AI-enabled cockpit systems create certification and verification challenges. Continual learning may change system behavior after qualification, complicating configuration control, traceability, and assurance. Operational systems may therefore require validated model versions, controlled update cycles, performance monitoring, rollback capability, and event logs rather than unconstrained in-mission self-modification. The evidentiary threshold should increase with the system’s authority and the consequence of an erroneous action.

Physiological and neurocognitive data are also unusually sensitive. They may reveal health conditions, fatigue, emotional state, or inferred readiness and could affect medical, occupational, or command decisions. Governance should specify purpose limitation, data minimization, access control, encryption, retention, secondary use, audit, and mechanisms for contesting an inference. A pilot-state model intended for real-time assistance should not automatically become an aeromedical diagnostic record or personnel-selection instrument.

Cybersecurity is inseparable from safety. Manipulated biosignals, corrupted aircraft-state data, adversarial inputs, or compromised external agents could provoke inappropriate adaptation or disclose operational vulnerability. The architecture should authenticate data sources, quantify sensor integrity, isolate critical functions, monitor anomalous inputs, and degrade safely when confidence is lost.

Ethical design also requires protection of agency and avoidance of automation-mediated coercion. Pilots should understand when physiological data are being used, what actions may follow, and which decisions remain contestable. Where operational necessity limits consent, the limitation and its governance require explicit justification and oversight. These safeguards are not peripheral constraints; they are conditions for trustworthiness and sustained adoption.

## 8 Limitations

This Perspective is intentionally forward-looking and does not validate a specific aircraft architecture, sensing suite, digital-twin implementation, or autonomy policy. Public descriptions of developmental military systems are incomplete and should not be treated as authoritative evidence of final capability. The term future fighter is therefore used instead of assigning the framework to a specific numbered generation.

Ecology is presently an organizing ontology and research heuristic, not a mathematically specified predictive theory. Its value will depend on whether the proposed entities, relationships, and propositions can be operationalized and tested. Several components may also be distributed across software and hardware rather than implemented as discrete modules. The PDT-IFMS separation is functional, not necessarily physical.

Finally, physiological inference in operational aviation remains constrained by signal quality, movement, environmental artifact, individual variability, construct ambiguity, and limited ground truth. A technologically sophisticated model can still be unsafe if it estimates the wrong construct, communicates certainty poorly, or triggers an ineffective response. The framework should therefore be treated as a research agenda, not an endorsement of immediate operational deployment.

## 9 Discussion

Future fighter aviation may evolve from interaction between a pilot and a collection of tools toward coordination within a distributed human-autonomy system. Established human-factors theories already explain many essential features of this transition. The pilot-wellness-informed ecological framework proposed here integrates those foundations with individualized physiological modeling, digital twins, adaptive autonomy, and longitudinal health considerations.

The central design principle is functional clarity. The PDT estimates and forecasts pilot state; the IFMS integrates pilot, platform, mission, and external information; the pilot retains intent, authorization, and override; and the aircraft executes commands within certified constraints. Pilot wellness becomes both an input to adaptation and an outcome by which the joint system is judged. The framework is deliberately testable and bounded by uncertainty, human authority, privacy, cybersecurity, and certification. Its success should be measured not by how much autonomy is added, but by whether the resulting team is safer, more predictable, more resilient, and better able to preserve pilot performance and long-term health.

## Data Availability

The original contributions presented in the study are included in the article/supplementary material. Further inquiries can be directed to the corresponding author.
